# Transcriptome analysis identifies genes regulating self-compatibility, flowering time, and oil biosynthesis in Noug (*Guizotia abyssinica*)

**DOI:** 10.1038/s41598-025-18728-x

**Published:** 2025-09-12

**Authors:** Adane Gebeyehu, Cecilia Hammenhag, Kassahun Tesfaye, Ramesh R. Vetukuri, Rodomiro Ortiz, Mulatu Geleta

**Affiliations:** 1 Department of Plant Breeding, Swedish University of Agricultural Sciences, P.O. Box 190, Lomma, 23422 Sweden; 2 Bio and Emerging Technology Institute, P.O. Box 5954, Addis Ababa, Ethiopia

**Keywords:** *De Novo* transcriptome assembly, Differentially expressed genes, Fatty acid, Photoperiod sensitivity, Self-compatibility, Plant sciences, Plant breeding

## Abstract

Noug (*Guizotia abyssinica*) is an economically important oilseed crop in Ethiopia that contributes significantly to local edible oil production and is a good protein source in animal feed. Despite its agronomic importance, the molecular basis of key agronomic traits, such as self-compatibility, photoperiod sensitivity, and oil biosynthesis, remains poorly understood due to the limited availability of genomic resources. To bridge this knowledge gap, we conducted extensive transcriptome profiling of 30 phenotypically diverse noug genotypes through RNA sequencing and *de novo* assembly. Our analysis generated 409,309 unigenes with an N50 of 584 bp, representing an extensive transcriptomic resource currently available for this crop. A total of 2,547 differentially expressed genes (DEGs) were identified, among which 409 were particularly associated with fatty acid metabolism pathways. Gene Ontology (GO) and Kyoto Encyclopedia of Genes and Genomes (KEGG) analyses revealed significant enrichment in lipid metabolism, stress response, and floral development pathways. Notably, many transcription factor families, such as bHLH, MYB, and WRKY, were differentially expressed between early- and late-flowering genotypes and high- and low-oil varieties, suggesting their regulatory roles in these traits. Transcriptome assembly revealed 58,852 putative transcription factors distributed in 51 families. This study provides fundamental genomic resources for marker-assisted breeding to improve productivity, oil quality, and stress resistance. The identified candidate genes present new opportunities for this underutilized yet agronomically valuable crop through modern biotechnological approaches.

**Supplementary Information:**

The online version contains supplementary material available at 10.1038/s41598-025-18728-x.

## Introduction

Noug (*Guizotia abyssinica*) is an economically important oilseed crop primarily cultivated in Ethiopia and India, contributing significantly to local edible oil production^1,2^. In addition to its value in human consumption, noug seeds are also used in the United States and Europe to feed birds, particularly finches. The crop is diploid with 2n = 30 chromosomes^3^ and it relies on a strict outcrossing reproductive method that heavily depends on honeybee pollination^4–6^. Despite its economic importance, where 30% of the country’s oilseed production and 26% of the produced oil are retained for home consumption^7^ it is economically less explored than other oilseeds, such as soybean or sunflower^8^. While its agronomic potential is evident^9^ a lack of genomic tools has been a constraining factor for molecular analysis of mechanisms regulating key traits such as self-compatibility, photoperiod sensitivity, and oil biosynthesis.

Unlike well-studied oil seeds such as sunflower, noug lacks a reference genome, and limited transcriptomic resources are available. Although it originated and has been domesticated in Ethiopia^10^ and molecular marker studies have confirmed its high genetic diversity within and among populations and its wild relatives^11–16^ functional genomic data are scarce. This is critical because honeybee pollination, strict outcrossing habits, and variability in fatty acid constitution make breeding difficult.

Molecular marker-based studies have confirmed the wide genetic base of this crop, which aligns with trait diversity in locally adapted landraces^6,17–19^. This diversity supports the potential for breeding programs targeting desirable traits such as oil content, fatty acid composition, self-compatibility, days to maturity, and photoperiod response. Noug seeds generally contain 25–56% oil by weight, with an average oil content of approximately 35% ^6,17–19^. Oleic acid (C18:1) and linoleic acid (C18:2) dominate noug oil, comprising more than 90% of its fatty acid profile^19,20^. Although linoleic acid enhances nutritional value, its high levels reduce oxidative stability, limiting shelf-life and food applications^21,22^. Conversely, a relatively high oleic acid content enhances thermal stability, making it favorable for high-temperature cooking and biodiesel use^19,23^.

Consequently, this study examined the transcriptomic variation underlying key agronomic traits in noug to identify candidate genes for marker-assisted breeding. Hence, RNA-seq analysis of 30 diverse genotypes was conducted with the following three primary objectives: (1) to generate the first comprehensive *de novo* transcriptome assembly for noug; (2) to identify DEGs associated with target traits, including self-compatibility, photoperiod sensitivity, and oil biosynthesis; and (3) to annotate metabolic pathways and transcription factors and metabolic pathways potentially associated with fatty acid metabolism and the stress adaptation response.

## Results

### Transcriptome sequencing and assembly

**Fig. 1:**
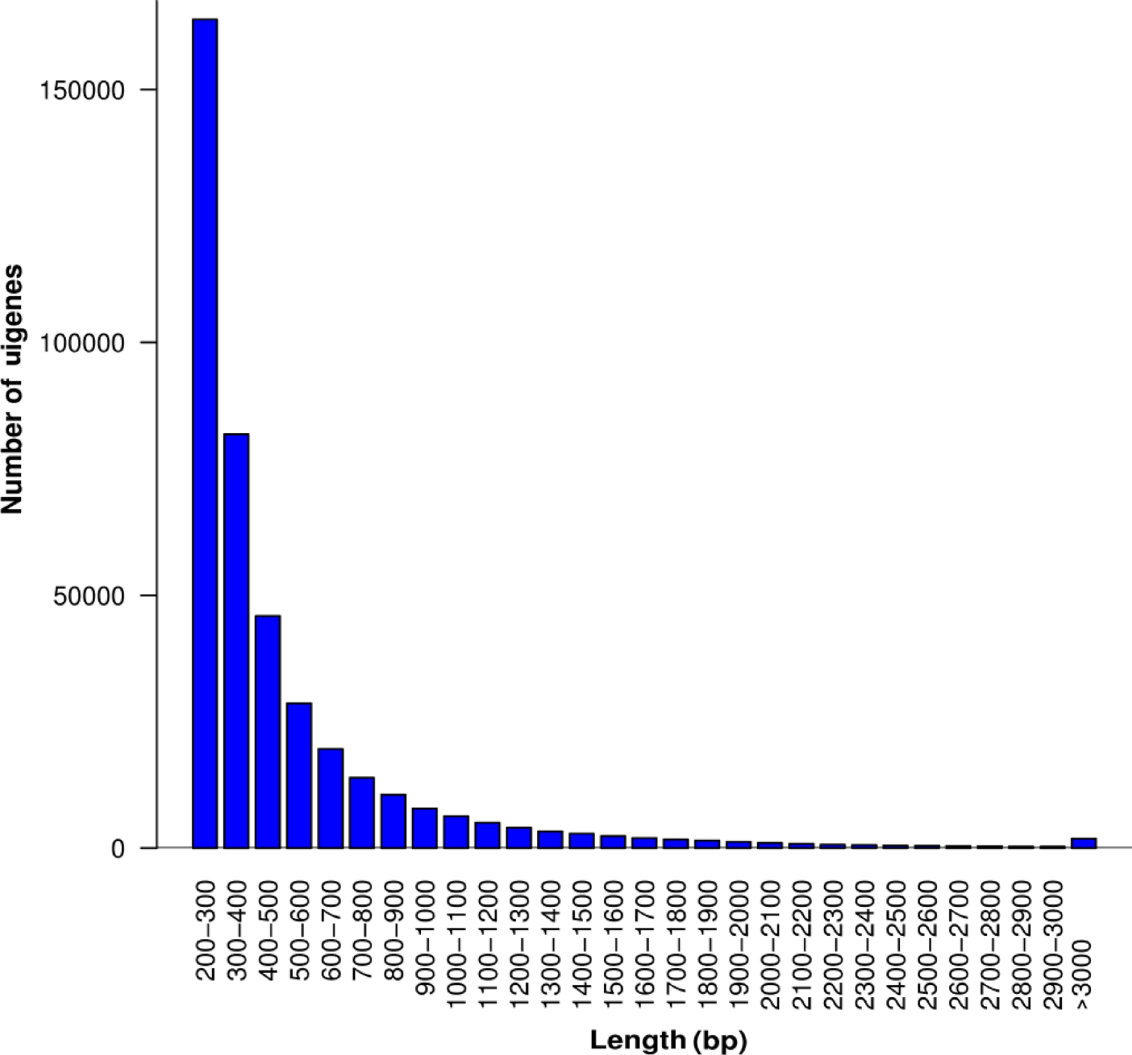
Unigene sequence length distribution. Unigene length is presented on the x-axis, whereas the number of unigenes in each range is given on the y-axis.

RNA-seq analysis generated 1.9 billion raw reads in 30 noug genotypes, with 64.5 million high-quality reads per genotype (Supplementary Table S1). Data quality assessment revealed that the average G + C content and Phred score of the raw reads met the quality criteria (G + C > 45%, Q30 > 94%, and average quality score > 36). Thus, the transcriptome dataset is considered suitable for downstream analysis of the transcriptome. Following stringent quality filtering (Phred score ≥ 30) and adapter trimming with Cutadapt v2.10, the resulting 1.82 billion clean reads were *de novo* assembled via Trinity v2.1.1 with default parameter settings (k-mer size = 25, min_contig_length = 200), resulting in 561,322 transcripts that coalesced into 409,309 unigenes after redundancy removal (Table 1). Length distribution analysis revealed a bimodal pattern, with many sequences (71.8%) falling within the range of 200–500 bp (403,196 transcripts) and a significant proportion of longer sequences (9.3% >1 kb, 10,609 contigs > 2 kb). The assembly showed robust metrics, including a maximum contig length of 13.6 kbp, a mean length of 497.9 bp, and an N50 of 590 bp, comparable to those of other oilseed crop transcriptomes. The N50 (584 bp) exceeds that of sunflower (390 bp), indicating robust assembly. While the unigene count (409,309) is high, it reflects noug’s heterozygosity and diversity, consistent with other complex de novo assemblies^24,25^. Putative TFs (58,852) were identified via strict PlantTFDB criteria (E-value < 1e-10, coverage > 50%, identity > 40%); fragmentation may inflate this estimate, requiring functional validation. The overall transcriptome assembly spanned 279.5 Mb, with comprehensive coverage of the expressed genome of noug (Fig. 1).

### Genotypic expression diversity and clustering patterns

**Fig. 2:**
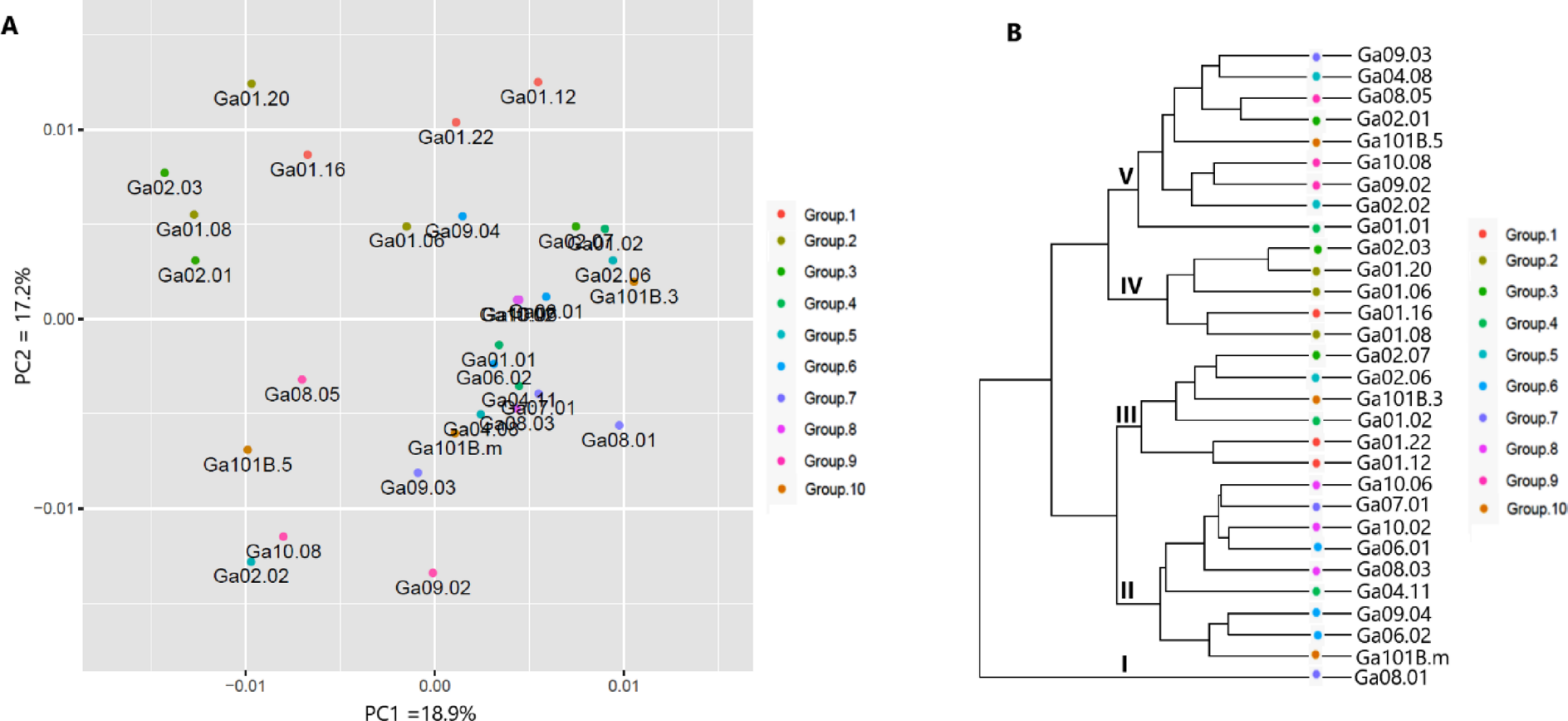
The overall gene expression-based cluster analyses depict the relationships among the 30 genotypes: (**A**) principal component analysis (PCA) scatter plot and (**B**) unweighted pair group method with arithmetic mean (UPGMA) dendrogram.

Principal component analysis of the normalized expression data for all 409,309 unigenes revealed biologically meaningful differences between genotypes. The first two principal components accounted for 36.1% of the total transcriptional variation (PC1: 22.4%; PC2: 13.7%), as depicted in Fig. 2A. UPGMA clustering based on Euclidean distances identified five dominant clusters (I-V) with distinct expression profiles (Fig. 2B). Specifically, Group-10 genotypes presented the most differentiated expression profiles, forming a separate cluster (II) with longer average lengths than the remaining groups. In contrast, Group-7 genotypes were heterogeneously classified into three distinct groups (I, II, V), suggesting strong underlying transcriptional plasticity despite phenotypic similarity. These patterns are consistent with previously reported genetic differences in noug populations^6,17–19^ and provide novel insights into the expression-level variation underlying agronomic traits. The observed transcriptional variation found in Groups 7 and 10 indicates the potential for selective breeding to capitalize on this natural variation.

**Table 1:** Summary statistics of the assembled Noug transcripts and unigenes.

Description	Transcripts		Unigenes	
	Count	Percent	Count	Percent
200–500 bp contigs	403,196	71.8	291,577	71.2
501–1000 bp contigs	105,702	18.8	80,467	19.7
1–2 kbp contigs	41,815	7.4	30,131	7.4
Above 2 kbp contigs	10,609	1.9	7,134	1.7
Total number of contigs	561,322	100.0	409,309	100.0
Maximum contig length (kbp)	13.6	-	13.6	-
Mean contig length (bp)	497.9	-	498.9	-
N50 contig length (bp)	590	-	584	-
Total number of bases in the contigs	279.5	-	204.2	-

### Comprehensive functional annotation

BLAST searches were conducted on 409,309 unigenes in six major databases. The multi-database annotation pipeline successfully assigned putative functions to 211,945 unigenes (51.8% of the total) with significant hits (E-value cutoff = 1e-5) in at least one of the databases, with detailed breakdowns shown in Table 2.

**Table 2:** The number and percentage of unigenes annotated via BLAST in six different databases.

Database	Count	Percent
GO	115,216	28.1
KEGG	151,713	37.1
NR	169,986	41.5
NT	160,406	39.2
PlantTFDB	58,852	14.4
UniProt	154,579	37.8
Unigenes annotated in one or more databases	211,945	51.8
Total number of unigenes	409,309	--

GO = Gene Ontology; KEGG = Kyoto Encyclopedia of Genes and Genomes; NR = nonredundant protein; NT = nucleotide; Plant TFDB = plant transcription factor database; UniProt = Universal proteins.

**Fig. 3:**
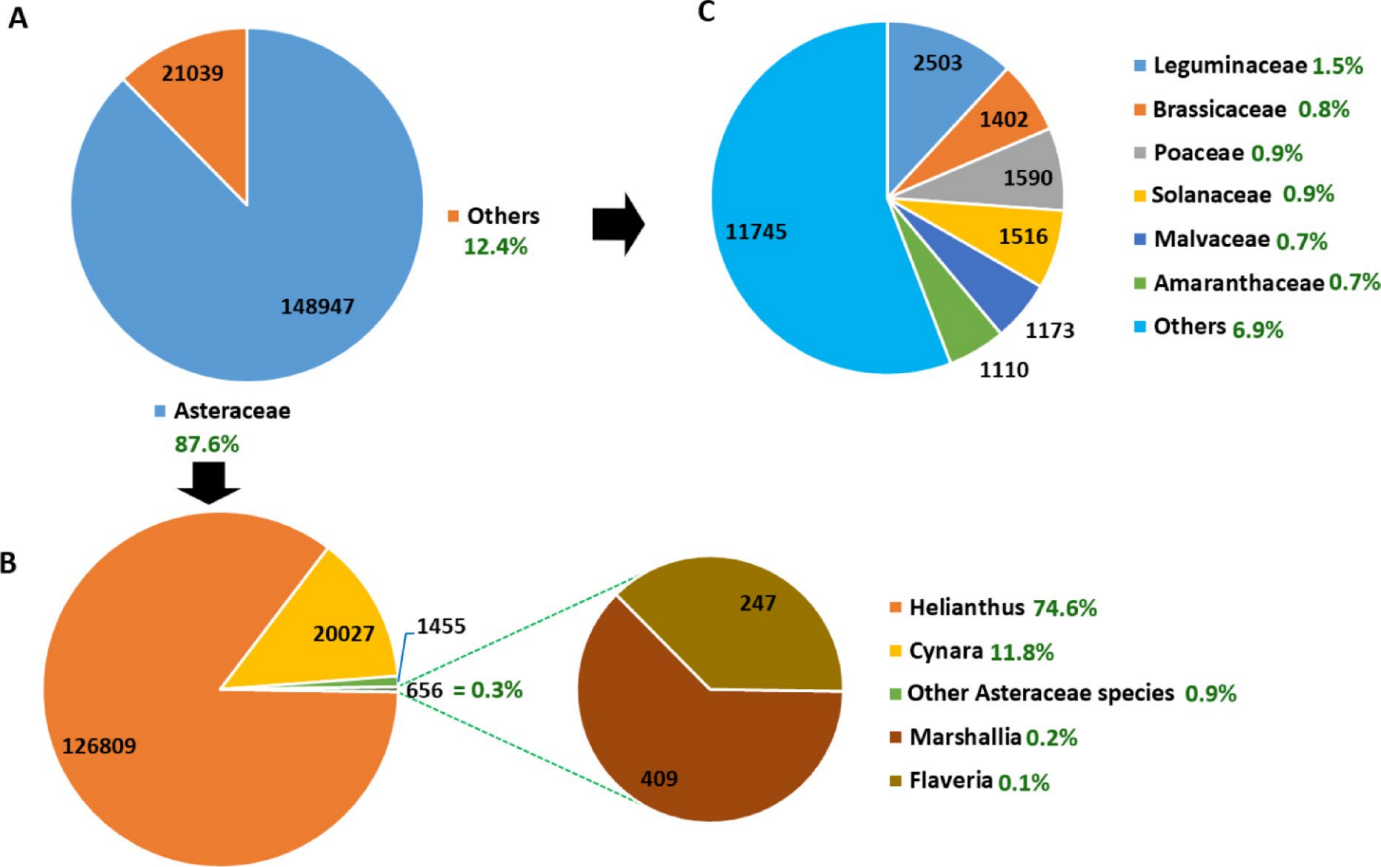
Pie charts displaying the number of unigenes that had significant BLAST hits against the sequences of Asteraceae and other plant families in the NCBI nonredundant protein database (NR): the number of unigenes annotated against Asteraceae species versus those annotated against species of other plant families; (**A**) further classification of the unigenes annotated against Asteraceae species; (**B**); further classification of the unigenes annotated against non-Asteraceae species (**C**). The percentage value beside each category indicates the proportion of unigenes annotated in the NR database for that category.

Among the unigenes annotated in the NR database, 87.6% had homology to protein sequences of the Asteraceae family, with *Helianthus*, *Cynara*, and other Asteraceae species accounting for 74.6%, 11.8%, and 1.2%, respectively (Fig. 3A and B). Among the unigenes with significant hits against *Helianthus* species, 99.9% were against *H. annuus*, reflecting their close phylogenetic relationship. The remaining 12.4% of the annotated unigenes were homologous to diverse plant families, including Leguminaceae (1.5%), Poaceae (0.9%), Solanaceae (0.9%), and Brassicaceae (0.8%) (Fig. 3A and C).

**Fig. 4:**
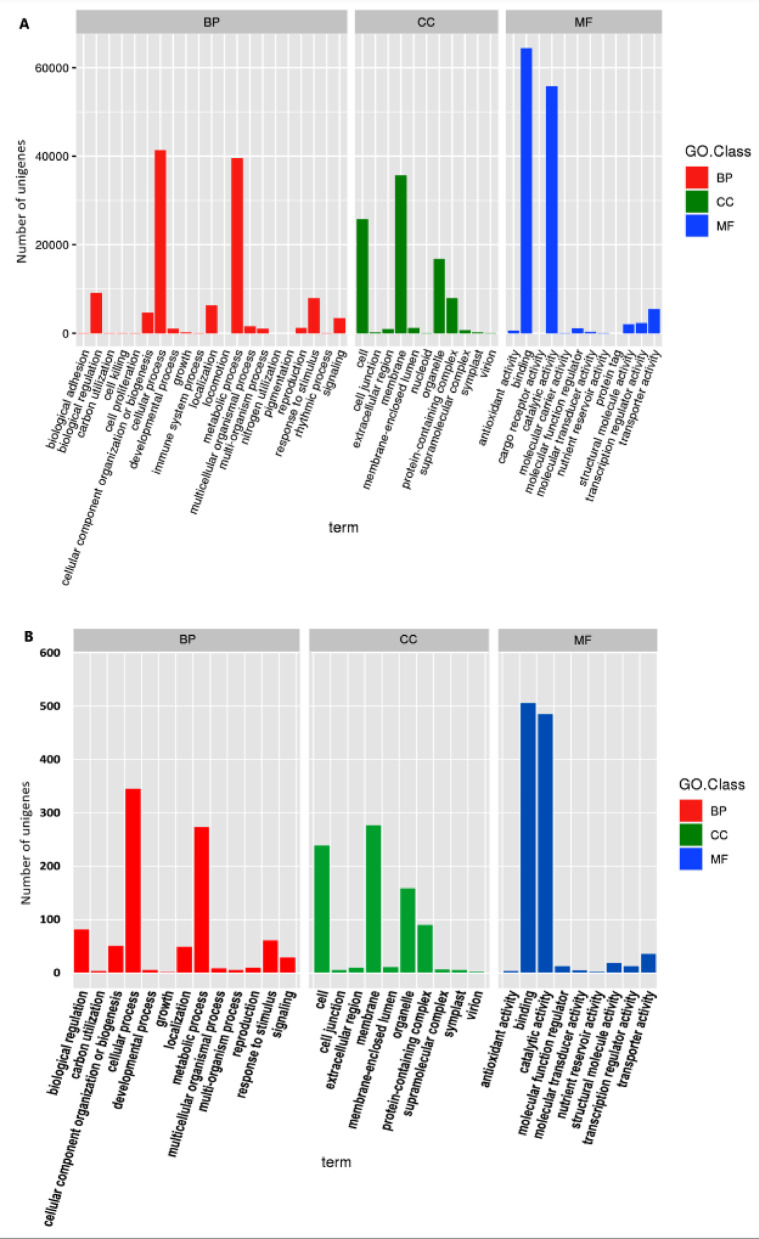
Gene Ontology (GO) annotations of noug (**A**) expressed genes and (**B**) significantly differentially expressed genes to different functional categories of biological process (BP), cellular component (CC), and molecular function (MF) GO classes.

**Fig. 5:**
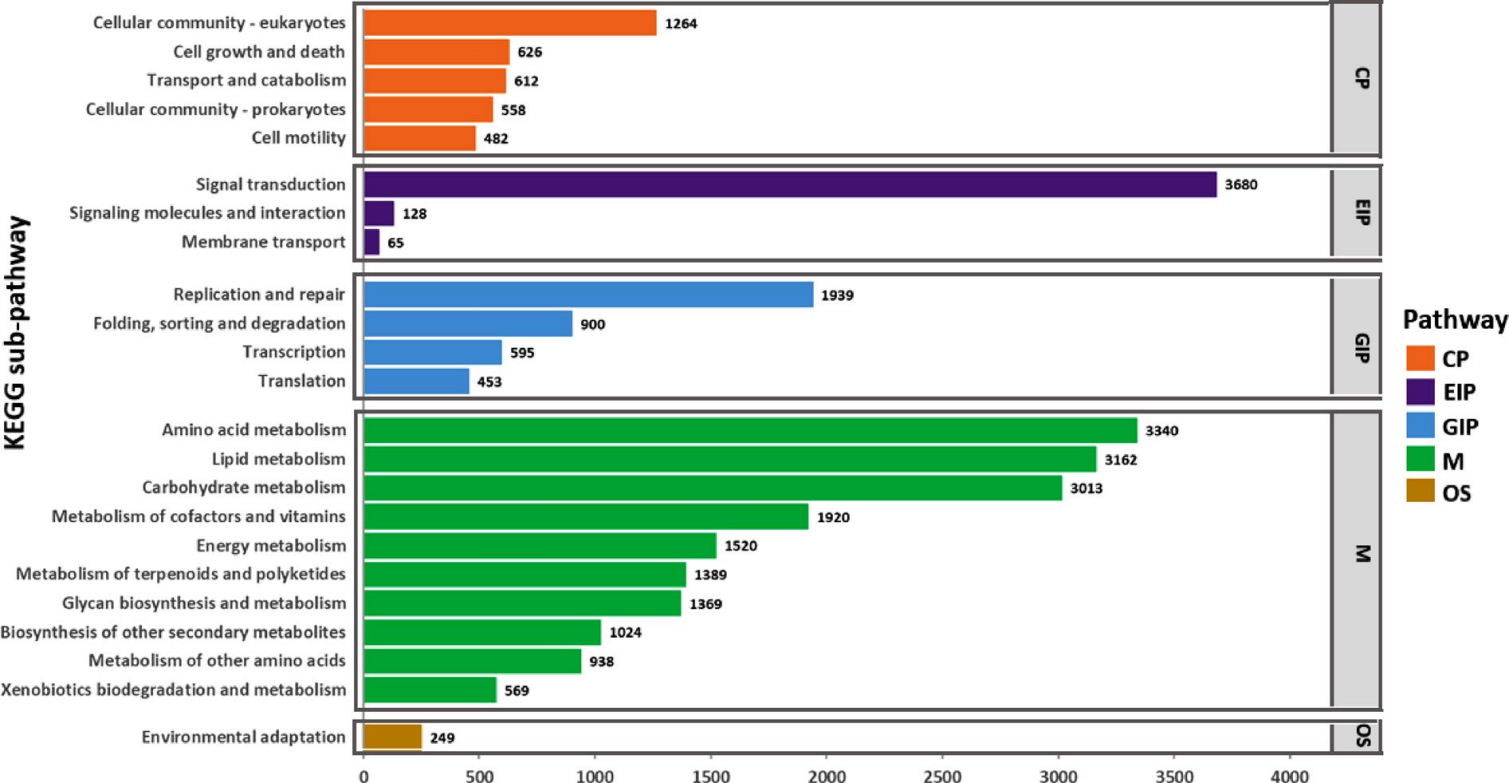
Kyoto Encyclopedia of Genes and Genomes (KEGG) annotation of noug unigenes into different sub-pathways of the five KEGG pathway classes: cellular processes, environmental information processing, genetic information processing, metabolism, and organismal systems.

Gene Ontology classification assigned 115,216 unigenes (28.1%) to three major categories: the biological process (BP), cellular component (CC), and molecular function (MF) classes (Fig. 4). The BP class was dominated by cellular (41,388) and metabolic (39,616) processes; the CC class was enriched for membrane (35,641), cell (25,795), and organelle (16,849) terms; and the MF class was predominantly associated with binding (64,432) and catalytic (55,812) activities. The KEGG pathway analysis mapped 29,795 unigenes (19.6% annotated) to 161 metabolic and regulatory pathways (Fig. 5), with higher representation of lipid metabolism (3,162 unigenes), signal transduction (3,680), carbohydrate metabolism (3,013), and amino acid metabolism (3,340).

The comprehensive TF analysis identified 58,852 putative transcription factors in the PlantTFDB spanning 51 families (Supplementary Figure S1), with bHLH (5,365; 9.1%), MYB-related (3,978; 6.8%), and LBD (3,888; 6.6%) being the most prevalent. These annotations provide crucial functional context for interpreting differential expression patterns.

There were significant matches between transcription factor genes (TFs) from 162 plant species and the unigenes, with lettuce (*Lactuca sativa*), radish (*Raphanus sativus*), and wild tomato (*Solanum pennellii*), being the top three, accounting for 4,015 (6.8%), 3889 (6.6%), and 3,579 (6.1%), respectively (Supplementary Figure S1). Only 612 unigenes (1.0%) were significantly associated with sunflower (*Helianthus annuus*) transcription factors.

### Differential gene expression analysis

Ten different pairwise comparisons were made to determine the DEGs and significant DEGs between each pair (Table 3). Pairwise comparisons were selected to contrast groups with divergent traits: Group-2 (reference: high self-seed set, intermediate flowering) vs. Groups 1,3,4,5,7 (varying self-compatibility/oil); Group-7 (very late flowering) vs. Group-8 (very early flowering) for maturity; and Group-7/8 (photoperiod-sensitive) vs. Group-10 (photoperiod-insensitive). Comparative expression analysis via DESeq2^6,17–19^ identified 2,330 DEGs with FDRs < 0.1, including 1,781 significant DEGs (FDR < 0.05, log_2_FC > 1) (Table 3; Supplementary Table S2). The number of significant DEGs varied substantially between group comparisons, ranging from 43 (Group-2 vs. Group-1) to 572 (Group-2 vs. Group-4).

**Table 3:** The number of DEGs and significant DEGs between the 30 genotypes and the 10 groups.

Group	DEGs total	DEGs up	DEGs down	Sig DEGs total	Sig DEGs up	Sig DEGs down
Genotypes (30)	2330	na	na	1781	na	na
Group-2 vs. Group-1	63	24	39	43	14	29
Group-2 vs. Group-3	211	90	121	175	80	95
Group-2 vs. Group-4	742	352	390	572	270	302
Group-2 vs. Group-5	501	269	232	366	199	167
Group-2 vs. Group-7	490	259	231	360	192	168
Group-7 vs. Group-6	84	39	45	69	33	36
Group-7 vs. Group-8	106	52	55	64	30	34
Group-7 vs. Group-9	127	38	89	98	27	71
Group-7 vs. Group-10	479	174	305	350	114	236
Group-8 vs. Group-10	590	228	362	450	164	286

DEGs up = upregulated differentially expressed genes; DEGs down = downregulated differentially expressed genes; Sig DEGs up = upregulated significantly differentially expressed genes; Sig DEGs down = downregulated significantly differentially expressed genes. DEGs adjusted *P* value < 0.1; log2FC< -0.5 or > 0.5 and Sig DEGs; adjusted p value < 0.05; log2FC< -1 or > 1).

**Fig. 6:**
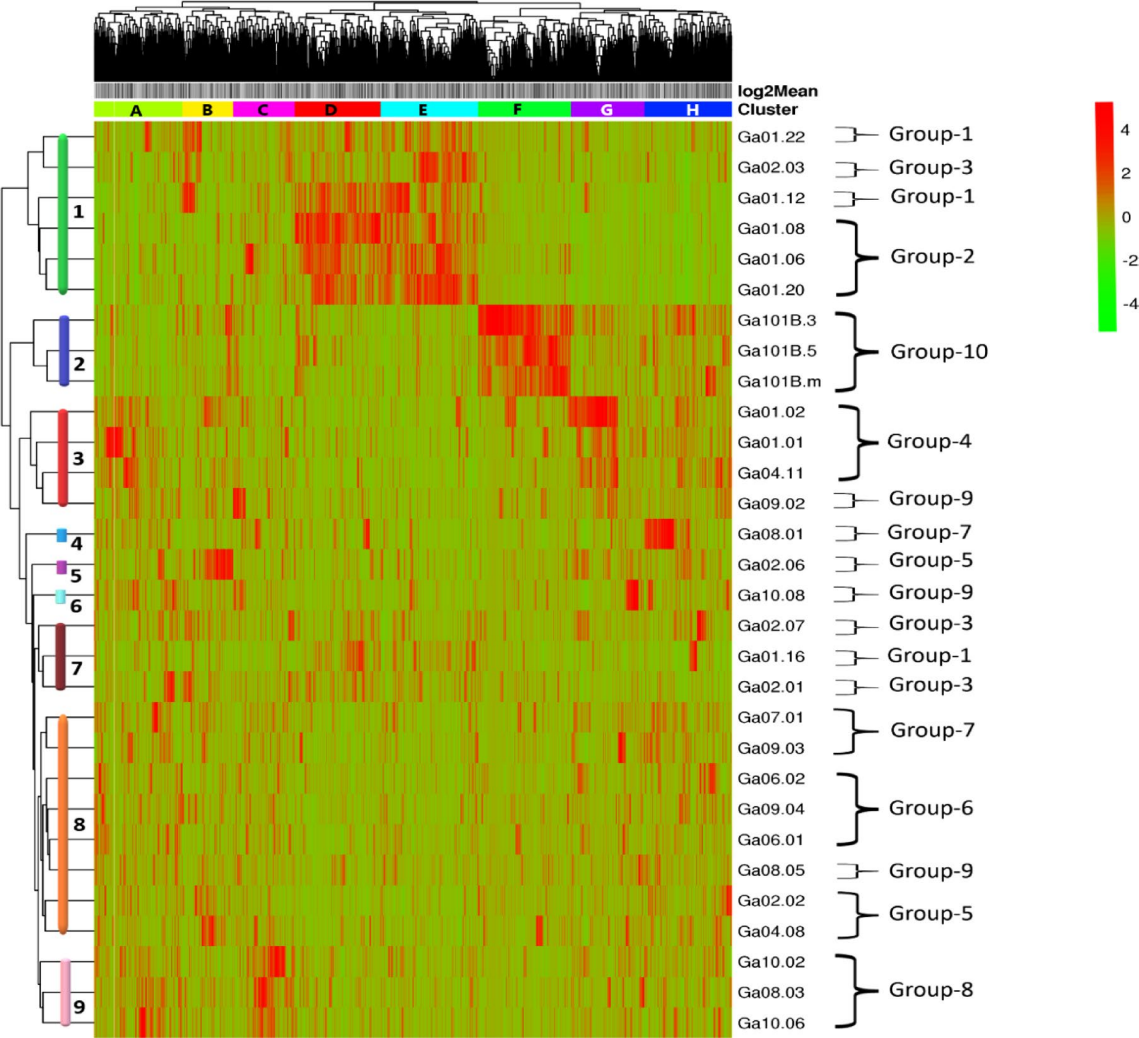
A heatmap depicting the expression patterns of 1,781 unigenes that were significantly differentially expressed among the 30 noug genotypes (forming eight clusters; clusters **A** to **H**), and the genotypes were grouped into nine clusters along the y-axis (Clusters 1 to 9).

Hierarchical clustering revealed eight distinct gene expression clusters (A-H) and nine genotype clusters (1–9) with characteristic patterns across genotypes (Fig. 6). Clusters D and E contained many genes whose expression was upregulated in the genotypes of Group-2 but downregulated in the genotypes of the other nine groups. A similar pattern was observed in cluster F, where many genes were upregulated in Group-10 but downregulated in the other nine genotypes. Notably, clusters D and E were upregulated in Group-2 (self-compatible), cluster F was specifically upregulated in Group-10 (photoperiod insensitive), and cluster G was downregulated in late-flowering genotypes.

**Fig. 7:**
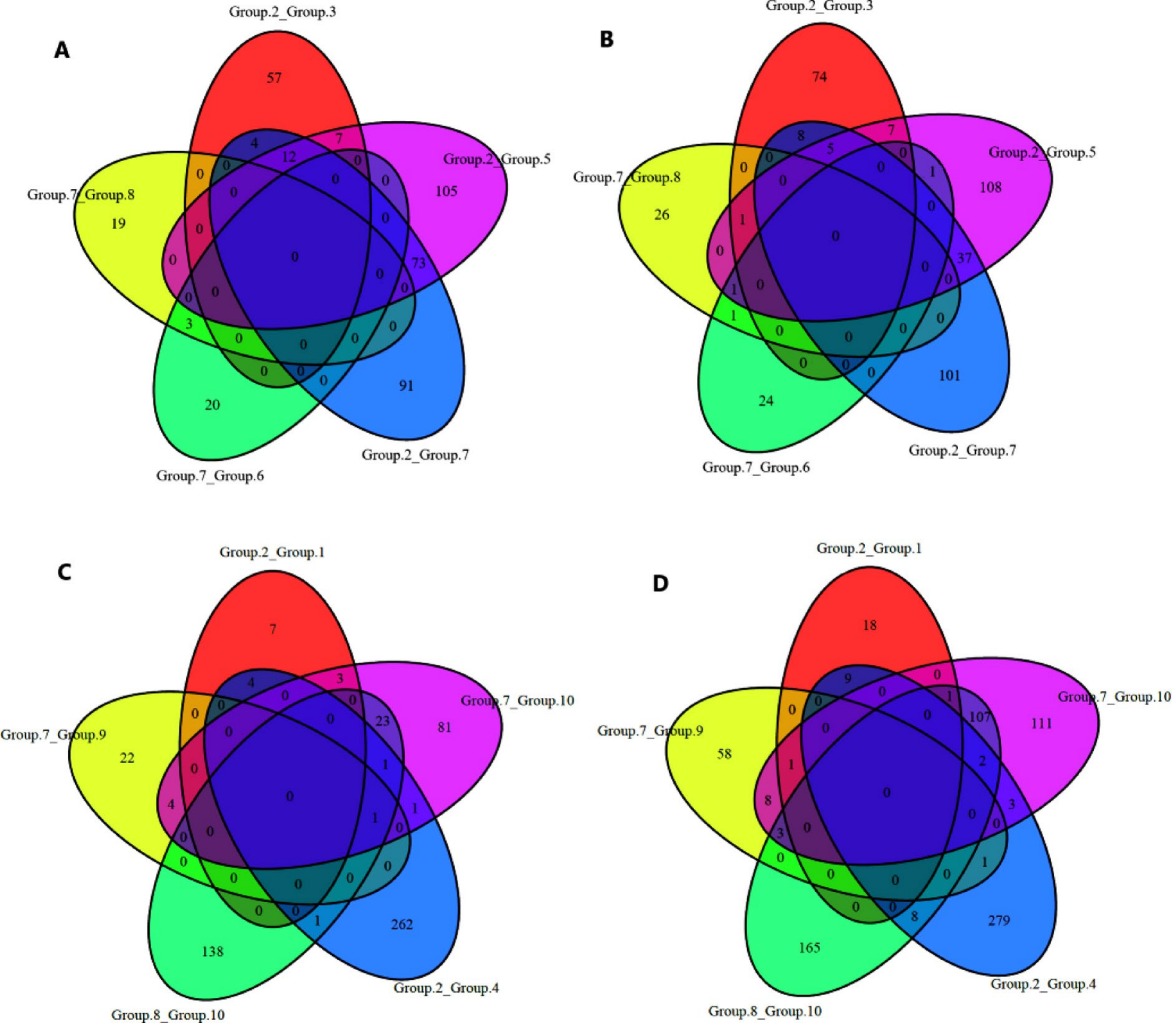
Venn diagrams illustrating the number of DEGs that were upregulated or downregulated across different compared group pairs: (**A**) comparing the first five group pairs (category-1) for upregulated genes; (**B**) comparing the first five group pairs (category-1) for downregulated genes; (**C**) comparing the second five group pairs (category-2) for upregulated genes; and (**D**) comparing the second five group pairs (category-2).

Venn diagram analysis (Fig. 7) revealed shared and unique DEG sets across trait comparisons, suggesting specialized and pleiotropic genetic regulation. The significant DEGs were also compared by grouping the ten pairs of groups (Table 3) into two categories, each with five pairs (Fig. 7).

**Fig. 8:**
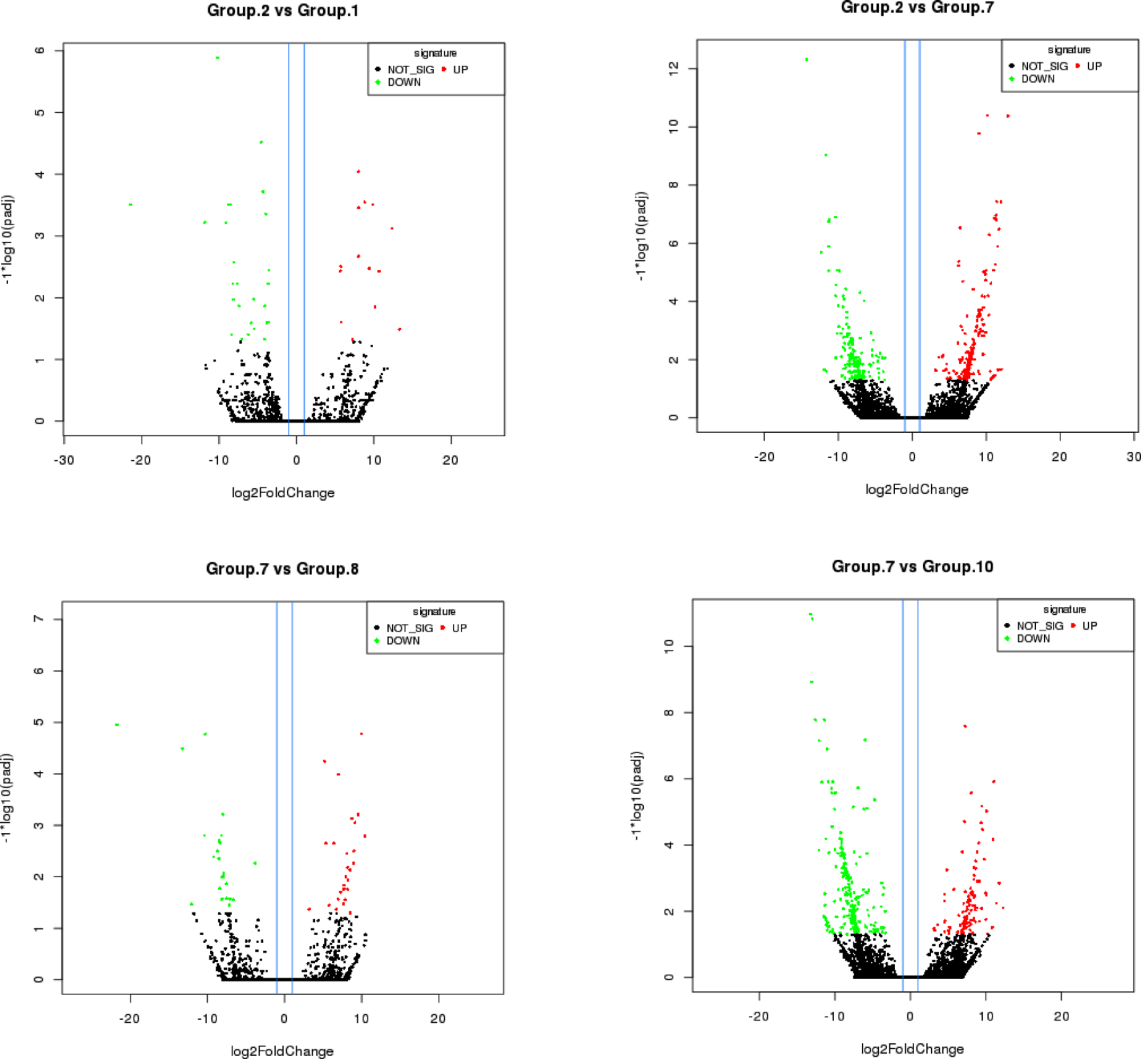
Volcano plots of significantly differentially expressed genes for (**A**) Group-2 vs. Group-1, (**B**) Group-2 vs. Group-7, (**C**) Group-7 vs. Group-8, and (**D**) Group-7 vs. Group-10. Each dot corresponds to a gene. In the two groups compared, dots in green denote upregulated genes, dots in red denote downregulated genes, and dots in black denote genes that were not significantly differentially expressed between the groups compared.

Volcano plot analysis highlighted several significant DEGs between Group-2 versus Group-1 and Group-7, Group-7 versus Group-8, and Group-10 (Fig. 8). Group-2 and Group-1 differ in self-seed set levels; hence, the significant DEGs between them include genes relevant to self-compatibility. The significant DEGs between Group-2 (intermediate flowering time) and Group-7 (very late flowering time) included those related to flowering time. Similarly, the significant DEGs between Group-7 (very late maturing) and Group-8 (very early maturing) included genes related to flowering time. The DEGs of Group-7 vs. Group-10 were related to flowering time and photoperiod sensitivity.

### Validation of differentially expressed genes

Eight candidate DEGs representing key traits were validated by qRT-PCR in 18 genotypes spanning four phenotypic groups. Strong concordance was observed between RNA-seq and qRT-PCR results (R²=0.89, *P* < 0.001; Supplementary Table S7). For example, TRINITY_DN97581_c0_g3_i2 (oil biosynthesis) showed consistent upregulation in high-oil genotypes (log_2_FC = 2.1, qRT-PCR ΔΔCt = -3.4 cycles; *P* = 0.003). The high correlation between log_2_FC and ΔΔCt values (R²=0.94, *P* < 0.001) confirms the reliability of our transcriptome analysis.

### Annotation of significantly differentially expressed genes

**Fig. 9:**
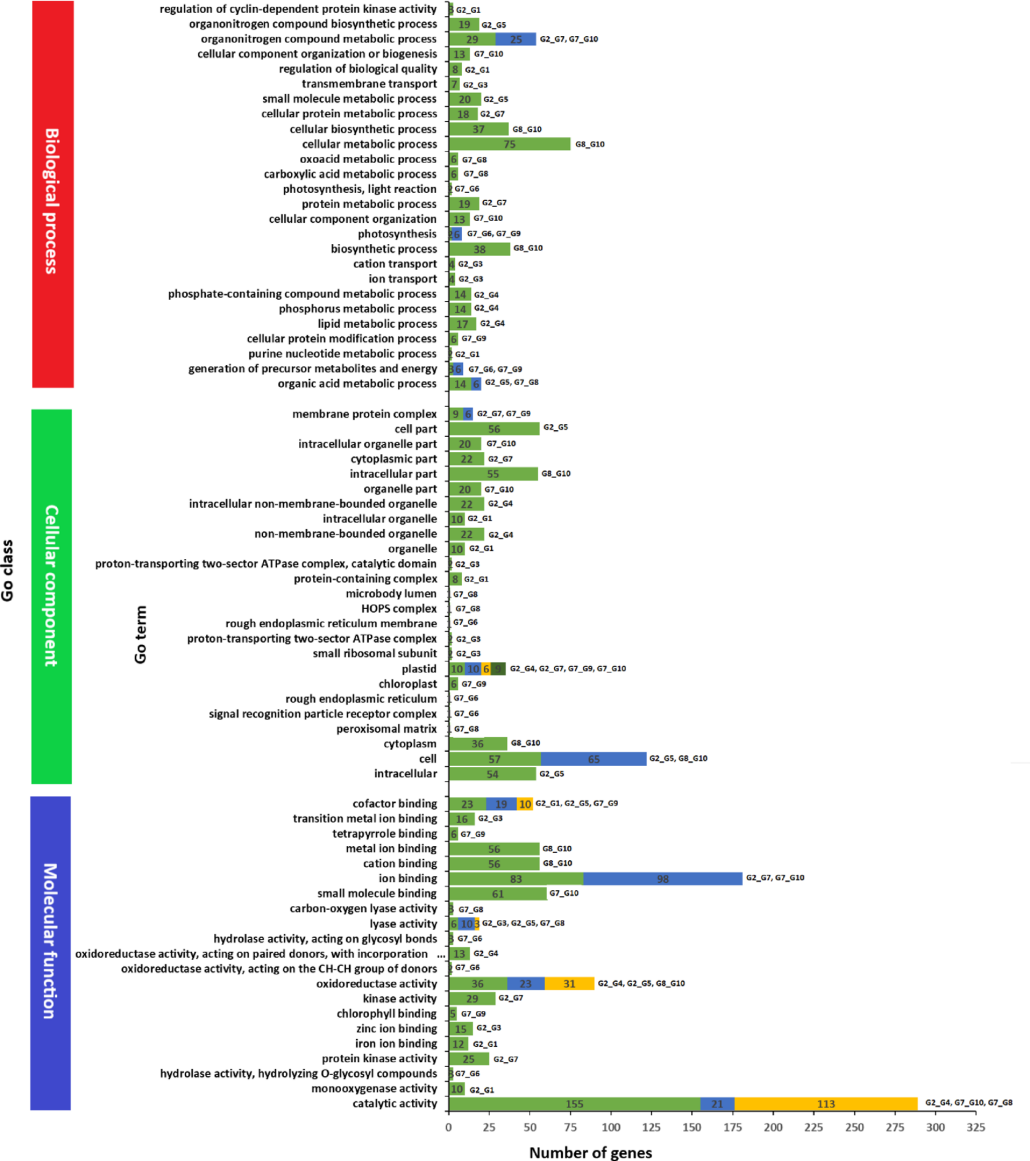
A horizontal bar graph of Gene Ontology (GO) annotations showing the number of genes significantly differentially expressed between pairs of groups to different GO terms of the biological process (BP), cellular component (CC), and molecular function (MF) GO classes. Each GO term corresponds to one to four group pairs. Note: Group pairs are abbreviated: e.g., G2_G1 refers to significant DEGs for Group-2 vs. Group-1.

**Fig. 10:**
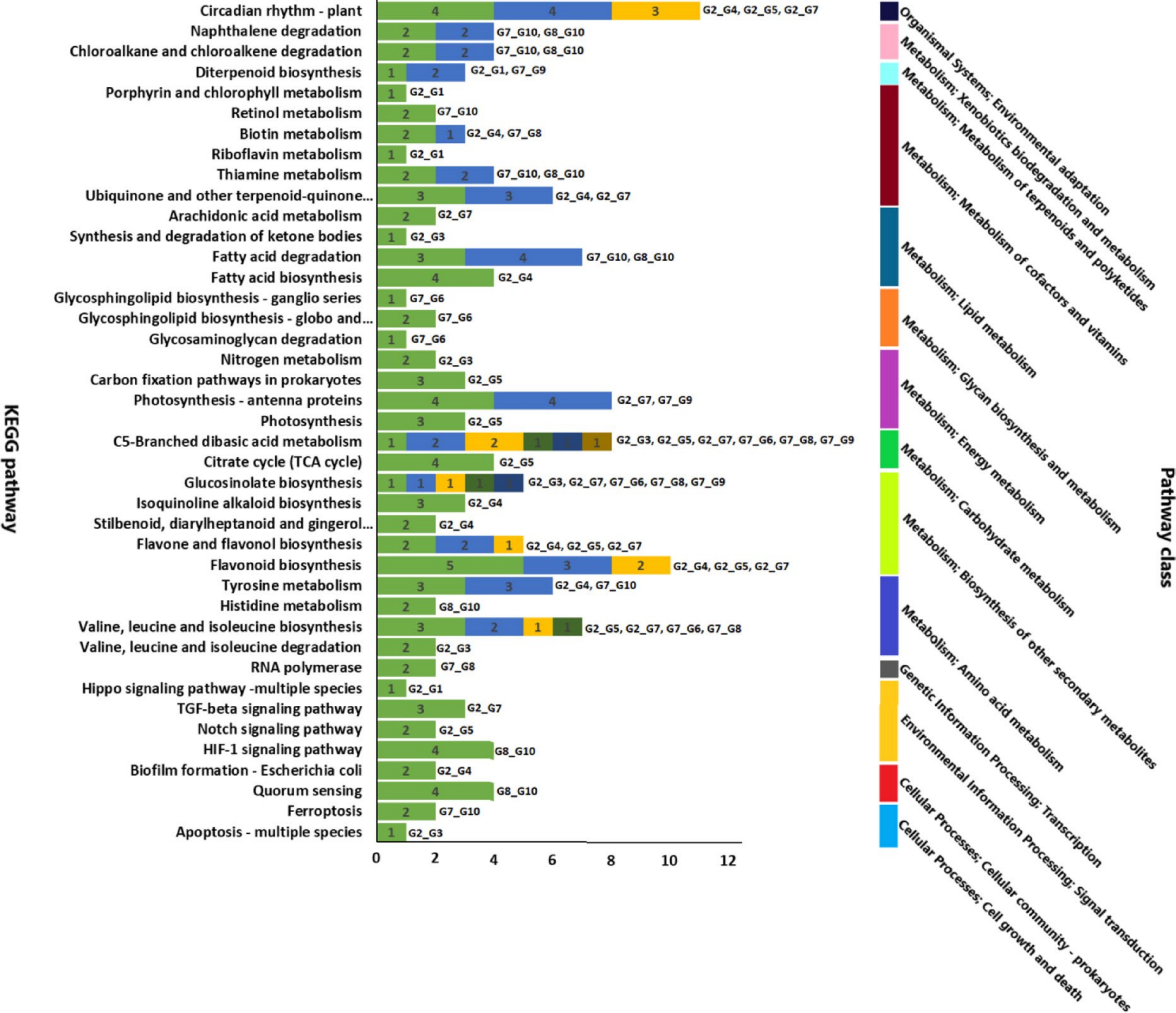
A horizontal bar graph of Kyoto Encyclopedia of Genes and Genomes (KEGG) annotations showing the number of significantly differentially expressed genes between pairs of groups to different enriched KEGG pathways of various KEGG pathway classes. Each KEGG pathway corresponds to one to six group pairs. Note: Group pairs are abbreviated: e.g., G2_G1 refers to significant DEGs for Group-2 vs. Group-1.

**Fig. 11:**
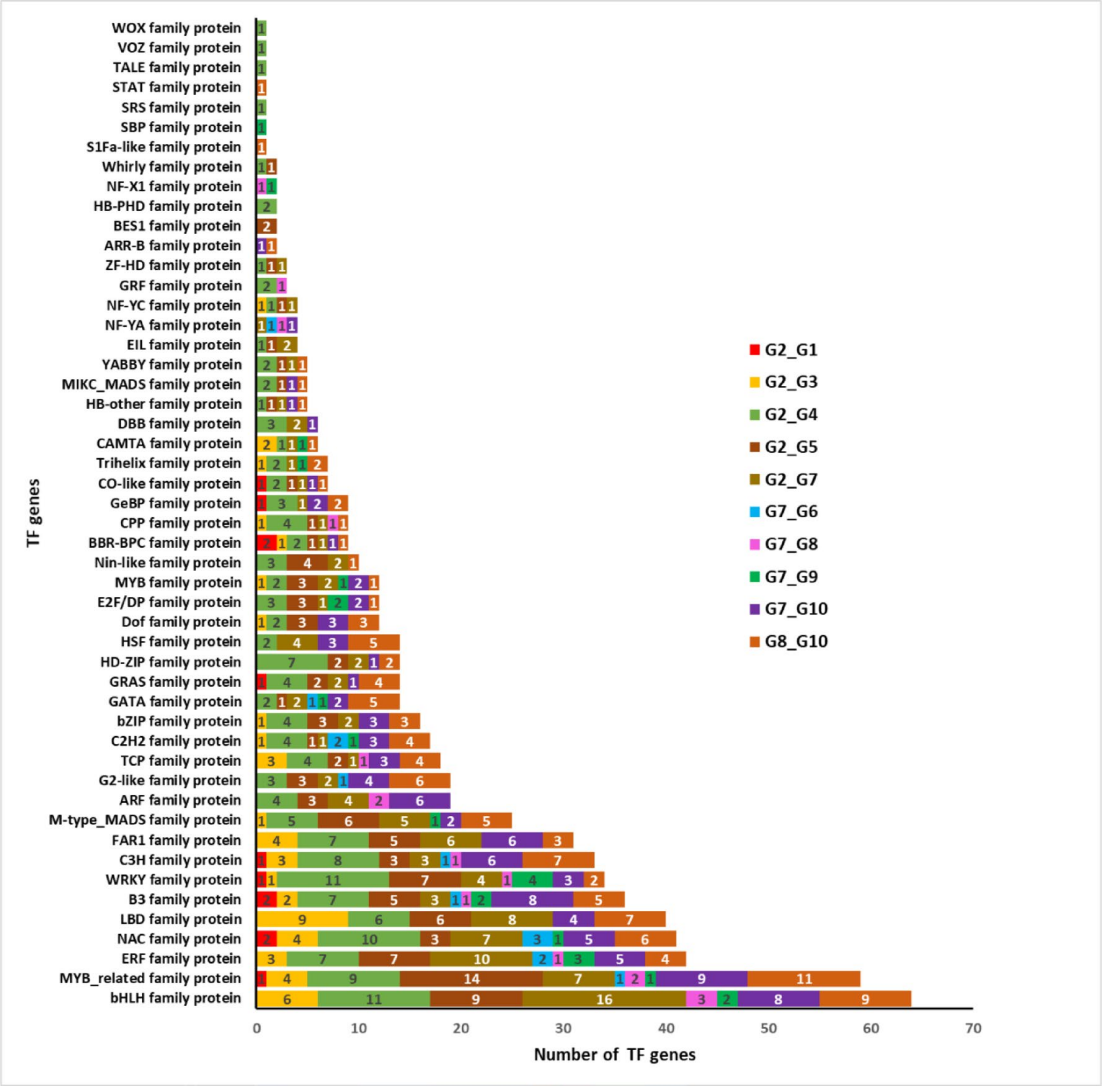
A horizontal bar graph showing transcription factors (TFs) corresponding to significantly differentially expressed genes between different pairs of groups. Each TF corresponds to one to ten group pairs. The number of significant DEGs for the corresponding group pair is given in the graph. Note: Group pairs are abbreviated: e.g., G2_G1 refers to significant DEGs for Group-2 vs. Group-1.

The annotation of significant DEGs between pairs of groups in different databases revealed their functional roles (Figs. 9, 10 and 11). The annotation in the GO database revealed many GO terms associated with significant DEGs between each pair of groups (Fig. 4B; Supplementary Table S4-A to J). The significant DEGs were annotated with 26, 25, and 21 terms from the BP, CC, and MF GO classes (Fig. 9), 148 DEGs across ten pairs of groups enriched for 41 KEGG pathways belonging to 14 KEGG pathway classes (Fig. 10; Supplementary Table S5-A to J), and 50 TF family proteins associated with significant DEGs across the ten group pairs (Fig. 11; Supplementary Table S6-A to J). The most frequent hit TF family was bHLH, whereas the most frequent hit plant species was *Lactuca sativa* (Fig. 11; Supplementary Figure S1).

### Significantly differentially expressed genes and self-compatibility

Four self-compatible (Group-1 to Group-4) and six self-incompatible genotypes were contrasted in this study (Table 4), with a focus on gene expression differences. Between Group-2 (high self-seed set) and Group-4 (very low self-seed set), considerable DEGs related to lipid metabolism, phosphorus metabolic processes, and plastid functions were present within the BP and CC categories of the GO. The MF category highlighted catalytic and oxidoreductase activities, with 155 and 36 DEGs, respectively (Fig. 9). Pathway analysis via the KEGG database revealed significant enrichment in four key metabolic processes associated with self-compatibility: flavonoid biosynthesis (ko00941), plant circadian regulation (ko04712), isoquinoline alkaloid production (ko00950), and terpenoid-quinone biosynthesis (ko00130). The TFs bHLH, WRKY, and NAC were overrepresented among the DEGs, suggesting their regulatory role in self-fertilization.

A comparative study of Group-2 (high self-seed set) and Group-1 (low self-seed set) identified DEGs involved in pollen and seed development, including genes involved in sporopollenin biosynthesis and pollen wall assembly. KEGG enrichment revealed riboflavin metabolism and diterpenoid biosynthesis (Fig. 10), whereas TF analysis revealed regulators such as BBR-BPC, NAC, and B3. Interestingly, these TFs were differentially expressed in Group-2 vs. Group-4, supporting the likelihood of their involvement in self-compatibility. Other comparisons between self-compatible (Group-2) and self-incompatible genotypes (Group-5 and Group-7) identified DEGs related to small molecule metabolism, protein kinase activity, and photosynthesis-related processes, with MYB-related, bHLH, and ARF TFs playing major roles.

This study revealed that self-compatible genotypes possess specific metabolic and gene expression profiles of regulatory genes compared with self-incompatible genotypes. The most important results are the involvement of flavonoid and terpenoid metabolic pathways, pollen development genes, and some TFs (e.g., bHLH, WRKY, NAC) in self-fertilization (Fig. 11). These findings constitute the foundation for studying the molecular mechanisms of self-compatibility, which can be directly translated to improve the reproductive efficiency of crops.

### Significantly differentially expressed genes and earliness

Concerning earliness, Group-7 (very late) is distinct from Group-8 (very early). The GO annotation of significant DEGs for Group-7 vs. Group-8 revealed that the most frequent terms were carboxylic acid metabolic process (GO:0000967), oxoacid metabolic process (GO:0097576), and organic acid metabolic process (GO:0000966) of the BP GO class, with six DEGs each (Fig. 9; Supplementary Table S4). Under the MF GO class, catalytic activity (GO:0003824) was the most frequent term, with 21 DEGs. KEGG annotation revealed that 15 of the 64 significant DEGs for this pair were related to 29 KEGG pathways (Supplementary Table S5). Five of these pathways, including RNA polymerase (ko03020), were enriched (*P* < 0.05). According to the PlantTFDB annotations, 16 of the 64 DEGs were annotated with 12 TF family proteins. The three most frequent DEGs were bHLH, ERF, and LBD, with 16, 10, and 8 DEGs, respectively (Fig. 11). NF-YA was the only TF that was differentially expressed between Group-4 and Group-5, but not between Group-2 and Group-4.

### Significantly differentially expressed genes and oil and oleic acid contents

On average, Group-1, Group-4, and Group-5 had higher oil and oleic acid contents than Group-2. The significant DEGs between Group-2 and Group-1 were annotated with 33 terms belonging to the MF GO class. Among these DEGs, TRINITY_DN82849_c0_g2_i2 was associated with diacylglycerol O-acyltransferase activity (GO:0004144) and acylglycerol O-acyltransferase activity (GO:0016411). However, other genotypes whose oil and oleic acid contents differ have inconsistent expression patterns for this gene. One of the nine TFs associated with significant DEGs between Group 2 and Group 1 was WRKY, which is known to regulate lipid biosynthesis.

Among the DEGs significantly upregulated in Group-2 compared with Group-4, TRINITY_DN97581_c0_g3_i2 was annotated with several terms of the three GO classes, including lipid metabolic process (GO:0006629; BP) and acyl-carrier-protein desaturase activity (GO:0045300; MF). Furthermore, this gene was upregulated in Group-2 compared with Group-5 and was annotated with several GO terms, including the fatty acid metabolic process (GO:0006631; BP). This DEG was annotated with pathways for fatty acid biosynthesis (ko00061) and unsaturated fatty acid biosynthesis (ko01040) in the KEGG database. Fatty acid biosynthesis was the second most enriched pathway for Group-2 vs. Group-4, which included four DEGs. The upregulation of the TRINITY_DN97581_c0_g3_i2 gene in high-oil genotypes suggests a role in lipid biosynthesis. Another DEG upregulated in Group-2 compared with Group-4 was TRINITY_DN105918_c1_g3_i1. This DEG was annotated with different KEGG pathways, including lipid metabolic process (GO:0006629; BP) and linoleic acid metabolism (ko00591). Among the TFs associated with the significant DEGs between Group-2 and Group-4 were bHLH, ERF, FAR1, MYB-related, and WRKY, which were previously reported to play a role in lipid biosynthesis.

### Significantly differentially expressed genes and photoperiod sensitivity

The photoperiod sensitivity was lower in Group-10 than in the other nine groups. Comparative transcriptome analysis of the photoperiod-sensitive (Group-7) and photoperiod-insensitive (Group-10) genotypes revealed that organonitrogen compound metabolism (GO:1901564; 25 DEGs), organelle components (GO:0044422; 20 DEGs), and catalytic activity (GO:0003824; 13 DEGs) were the highly enriched GO terms in the BP, CC, and MF classes, respectively (Fig. 9). Similarly, the GO annotation of significant DEGs between Group-8 (high photoperiod sensitivity) and Group-10 (low photoperiod sensitivity) revealed that cellular metabolic process (GO:0044237), cell (GO:0005623), and metal ion binding (GO:0046872) were the most frequent terms associated with the BP, CC, and MF GO classes, respectively, and occurred in 75, 65, and 56 significant DEGs (Fig. 9).

The KEGG annotation revealed that 56 significant DEGs between Group-7 and Group-10 belong to 64 KEGG pathways (Supplementary Table S5). Among these, seven pathways, to which 16 DEGs were assigned, were enriched (*P* < 0.05), including tyrosine metabolism (ko00350) and fatty acid degradation (ko00071). For Group-8 versus Group-10, 85 significant DEGs were attributed to 87 KEGG pathways, including circadian rhythm-plant (ko04712). Seven pathways were enriched, to which 20 DEGs were assigned (*P* < 0.05). These include quorum sensing (ko02024) and fatty acid degradation (ko00071) (Supplementary Table S5; Fig. 10). Among the significant DEGs between Group-7 and Group-10, 98 were associated with 30 different TFs. The three genes with the greatest frequency were MYB-related, bHLH, and B3, with 9, 8, and 8 DEGs, respectively (Fig. 11). In the case of Group-8 vs. Group-10, 110 DEGs were successfully annotated with 34 different TFs. The three most frequent TFs were MYB-related, bHLH, and C3H and included 11, 9, and 7 DEGs, respectively (Fig. 11).

## Discussion

RNA sequencing-based transcriptome profiling has emerged as a powerful tool for identifying differentially expressed genes (DEGs) in plant species, providing critical insights into their regulatory processes and functional implications. While extensively utilized in model organisms such as Arabidopsis^26^ brassicas^27,28^ and sunflower^29^ no complete transcriptomic analysis has yet been described for noug (*Guizotia abyssinica*). Our study fills this gap by characterizing gene expression profiles in noug genotypes, where priority has been assigned to critical agronomic traits such as self-compatibility, oil content, fatty acid composition, days to flowering, and photoperiod sensitivity. Identifying novel unigenes and DEGs further enriches genomic tools and resources for noug and underscores the need for functional validation to elucidate their roles in trait regulation. Although phenotypic groups (Table 4) did not always form distinct clusters in overall expression profiles (Fig. 2B), DEG analysis between groups contrasting for specific traits (e.g., SC vs. SI, early vs. late) successfully identified candidate genes. This suggests trait regulation involves specific transcriptional subnetworks rather than genome-wide shifts. These findings lay a foundation for molecular breeding strategies focused on developing improved cultivars with optimized traits.

### Transcriptome assembly and functional annotation

The N50 value, a key metric for assessing transcriptome assembly quality, was significantly greater in *G. abyssinica* (590 bp) than in *H. annuus* (390 bp)^30^ indicating a robust assembly^31–33^. Among the 409,309 unigenes identified, 51.8% (211,945) exhibited significant homology to sequences in public databases (E-value ≤ 1e-5). However, 58.5% lacked matches in the NR database, potentially due to their noncoding nature, short sequence length, or the limited availability of *G. abyssinica* genomic data. Among the annotated unigenes, 74.6% aligned with *H. annuus* proteins, whereas only 0.9% matched Asteraceae family proteins, highlighting the underrepresentation of noug in existing databases.

Gene Ontology (GO) analysis annotated 28.1% (115,216) of the unigenes into at least one GO term categorized as biological process (BP), molecular function (MF), or cellular component (CC) categories. Predominant BP terms included cellular and metabolic processes, biological regulation, and response to stimuli, whereas CC terms were enriched in membrane-, cell-, and organelle-related functions. MF annotations were dominated by binding, catalytic, and transporter activities, which is consistent with roles in signal transduction and metabolic regulation^34^. Further pathway analysis via KEGG revealed that 19.6% (29,795) of the annotated unigenes participated in 161 metabolic and regulatory pathways, with significant representation of lipid, amino acid, and carbohydrate metabolism, which aligns with findings in *H. annuus*^33^.

### Trait-associated gene expression patterns

Seed setting is a critical developmental stage regulated by genetic and environmental factors that affect seed number, size, and yield potential. In our study, many unigenes were linked to metabolism pathways with significant involvement in lipid, phosphorus, and phosphate-containing compound metabolic processes. Notably, E3 ubiquitin-protein ligases, known to regulate seed development^35^ were implicated in our dataset. Differentially expressed gene analysis revealed two important genes associated with this trait: DN97095_c2_g1_i7, a CBL-interacting serine/threonine-protein kinase 23 homolog (implicated in ATP binding and protein phosphorylation), and DN79699_c0_g3_i1, a putative guanosine tetraphosphate diphosphokinase RSH1 ortholog of *H. annuus* involved in nucleotide metabolism. These findings align with studies on the mechanisms of seed setting in *Brassica napus*^36^ suggesting conserved regulatory mechanisms.

Early maturity is another adaptive trait for drought escape in arid climates^37^. Early flowering in Arabidopsis is controlled by complex signaling networks of transcription factors (TFs) and metabolic alterations^35,38^. Fatty acids play a role in the synthesis of suberin and cutin wax to reinforce cell membrane integrity and the structural barrier against abiotic stresses^39^. Our findings revealed that the RNA polymerase IV pathway (ko03020) was significantly enriched in early-maturing genotypes. RNA Polymerase IV also takes part in pollen development in *Brassica rapa*^40^ where its activity during meiosis influences pollen formation^41^ and microspore development in *Capsella rubella*^42^ suggesting its role in accelerating reproductive development. Functional studies in *Capsella rubella* have shown that the loss of function of RNA polymerase IV disrupts microspore development^42^ indicating a direct mechanistic link between flowering time regulation and pollen development. These findings suggest that Pol IV-mediated epigenetic control of reproductive development may be responsible for the early-mature phenotypes observed in noug. Notably, these findings highlight the importance of RNA polymerase IV-mediated regulation to control flowering time adaptation in noug. In addition, bHLH, ARF, and MYB-related TFs were differentially expressed in the early-maturing genotypes. ARFs control auxin-responsive gene expression and influence developmental timing^43^.

Seed oil accumulation is an essential trait that involves *de novo* fatty acid synthesis in plastids and triacylglycerol (TAG) biosynthesis and assembly, with lipid degradation modulating energy homeostasis in the endoplasmic reticulum^44^. Fatty acid degradation, through lipolysis, produces TAG and generates metabolites such as acyl-CoA and acetyl-CoA via β-oxidation, which conserves energy^44^. Some genotypes have a low oil content because of the frequent degradation of fatty acids. Some of the most upregulated genes in our study were *DN46215_c0_g1_i1*, the lipid binding upregulated gene, and *DN98334_c1_g2_i2*, the acyl group transferase activity upregulated gene. Some of the genotypes are related to lipid transport and the oil content in the fatty acid degradation pathway (ko00071). MYB-related, bHLH, C3H, and LBD are the most dominant TF families involved in the regulation of developmental processes as well as metabolism, such as seed size and oil content, in *Brassica rapa*^45,46^. Upregulated genes such as *DN46215_c0_g1_i1* (lipid binding) and *DN98334_c1_g2_i2* (acyltransferase activity) suggest genotype-specific variations in oil content. The fatty acid degradation pathway (ko00071) was prominent among the DEGs, with MYB-related, bHLH, C3H, and LBD TFs playing central roles. MYB TFs, known to regulate lipid metabolism in green algae^47^ may similarly influence oil biosynthesis in noug. Hence, knowledge of fatty acid biosynthesis and degradation pathways is important in exploring the molecular mechanisms governing the oil content of noug. Crossbreeding genotypes with photoperiod-sensitive, self-incompatible, and high-oil-containing traits (e.g., Ga08-03 and Ga10-06) could yield photoperiod-insensitive, early-maturing cultivars with high seed and oil yields that are suitable for low-altitude cultivation. Further investigation of significant DEGs and TFs will significantly clarify the molecular mechanisms underlying genomic-led breeding for noug.

Photoperiod sensitivity is a critical trait that can be included in the environmental adaptations of plants. However, such adaptation through modification by DNA methylation is heritable, although reversible, and is individually dependent on external stress and developmental stimuli^48^. For example, IDM1 prevents DNA hypermethylation of homologous genes under stress to increase photoperiod sensitivity through IDM1 activities^49^. Two-component response regulator-like APRR3 is another process that regulates the photoperiodic flowering response in *Arabidopsis thaliana*^50^*Cicer arietinum*^51^ and *Glycine max*^52^. In addition, E3 ubiquitin-protein ligases are critical regulators of several pathways related to photoperiodism, mediating light responses through photoreceptors, phytohormones, and other signaling networks^53^. DEG analysis between photoperiod-sensitive and photoperiod-insensitive genotypes revealed that an environmental adaptation-associated gene, *DN94708_c2_g1_i11*, was significantly upregulated in photoperiod-insensitive genotypes. In addition, the dominant TF families, MYB-related, bHLH, C3H, and LBD, are involved in light signaling and stress responses. MYB-related TFs are most important in plant development and metabolic processes and modulate cell differentiation, the cell cycle, and hormone and environmental responses^54,55^. MYB and C3H TFs regulate *CONSTANS* and *FLOWERING LOCUS T* expression^56,57^ whereas LBD TFs increase drought tolerance^58^. This increased photoperiod insensitivity in the Group-10 genotype was presumably a consequence of more rapid induction of MYB-related, C3H, and LBD TFs triggered by environmental cues. Hence, crossbreeding photoperiod-insensitive genotypes (e.g., Ga08-03 and Ga10-06) with high-oil, self-compatible lines could yield early-maturing cultivars suitable for low-altitude cultivation.

## Conclusion

Our study presents the first comprehensive transcriptomic analysis of noug (*Guizotia abyssinica*), identifying 409,309 unigenes and 2,547 DEGs linked to key agronomic traits. Functional analyses revealed enriched pathways related to lipid metabolism and stress response, with bHLH, MYB, and WRKY transcription factors emerging as critical regulators. Notably, E3 ubiquitin ligases, RNA polymerase IV, and CONSTANS-like TFs were associated with flowering time and oil biosynthesis, suggesting targets for breeding climate-resilient cultivars. While 58.5% of the unigenes remain unannotated, this study lays a foundation for future functional studies (e.g., CRISPR-Cas9) and marker-assisted breeding to enhance noug productivity and stress adaptation.

## Materials and methods

### Plant material

This study utilized 30 phenotypically distinct noug genotypes (Table 4). Comprehensive phenotyping data (means, variances, statistical analyses) and trait images are published in^6,17–19^. With approximately two-thirds derived from breeding populations selected for improved traits, including self-compatibility, early maturation, reduced photoperiod sensitivity, and increased oil/oleic acid content, as described in^59^. Breeding populations were derived from crosses between Ethiopian landraces (detailed origins in^59^. Landraces were obtained from the Ethiopian Biodiversity Institute (accession numbers in Table S1 of^59^. Parents are not included; this study focuses on advanced/segregating material. One-third of the genotypes were selected from landrace populations based on differences in one or more target traits between them and from the breeding populations. Among the 30 genotypes, twelve are self-compatible, although to varying degrees, whereas the other eighteen are strictly self-incompatible. The days to maturity of the genotypes ranged from 120 days (very early types) to 180 days (very late types). Among the 30 genotypes, three were selected from breeding populations capable of flowering when the photoperiod exceeded 12 h. The mean oil content of the source populations was 30–45% of their dry seed weight. The oleic acid content of all the source populations except Ga01-16, Ga02-01, Ga01-02, and Ga02-02 was lower than 13%, although the oleic acid content primarily depends on the environmental temperature. The 30 genotypes were grouped into ten groups based on their similarity in one or more target traits described in Table 4 below.

The 30 genotypes were grouped into ten groups based on their similarity in one or more target traits described in Table 4 below. Grouping was based solely on phenotypic similarity in target traits (Table 4) to enable focused DEG analysis between trait extremes (e.g., high vs. low self-seed set), reducing complexity despite imperfect clustering in overall transcriptomes (Fig. 2B).

**Table 4:** Plant material (genotypes) used for this study and their general description.

Group	Genotype code	Source	Self-compatibility	Level of self-seed set	Earliness	Oil content (% )	Oleic acid content (% )	Photoperiod insensitivity
1	Ga01-12*	Breeding population for increased oil content	Yes	Low	Early	> 40	< 13	No
1	Ga01-16*	Breeding population for increased oleic acid content	Yes	Low	Medium	> 40	> 13	No
1	Ga01-22	Breeding population for increased oil content	Yes	Low	Medium	> 40	< 13	No
2	Ga01-06	Breeding line for self-compatibility	Yes	High	Medium	35–40	< 13	No
2	Ga01-08	Breeding line for self-compatibility	Yes	High	Medium	35–40	< 13	No
2	Ga01-20	Breeding population for increased oil content	Yes	High	Medium	> 40	< 13	No
3	Ga02-01	Breeding population for increased oleic acid content	Yes	Medium	Medium	> 40	> 13	No
3	Ga02-03	Breeding population for increased oil content	Yes	Medium	Late	> 40	< 13	No
3	Ga02-07	Breeding population for increased oil content	Yes	Medium	Medium	> 40	< 13	No
4	Ga01-01	Breeding population for increased oil content	Yes	Very Low	Medium	> 40	< 13	No
4	Ga01-02	Breeding population for increased oleic acid content	Yes	Very Low	Medium	35–40	> 13	No
4	Ga04-11	Breeding population for increased oil content	Yes	Very Low	Medium	> 40	< 13	No
5	Ga02-02	Breeding population for increased oleic acid content	No	None	Late	35–40	> 13	No
5	Ga02-06	Breeding population for increased oil content	No	None	Medium	> 40	< 13	No
5	Ga04-08	Breeding population for increased oil content	No	None	Medium	> 40	< 13	No
6	Ga06-01	High oil content landrace population	No	None	Early	> 40	< 13	No
6	Ga06-02	High oil content landrace population	No	None	Early	> 40	< 13	No
6	Ga09-04	High oil content landrace population	No	None	Early	> 40	< 13	No
7	Ga07-01	High oil content landrace population	No	None	Very Late	> 40	< 13	No
7	Ga08-01	Low oil content landrace population	No	None	Very Late	< 35	< 13	No
7	Ga09-03	High oil content landrace population	No	None	Very Late	> 40	< 13	No
8	Ga08-03	Breeding population for increased oil content	No	None	Very Early	> 40	< 13	No
8	Ga10-02	Low oil content landrace population	No	None	Very Early	< 35	< 13	No
8	Ga10-06	High oil content landrace population	No	None	Very Early	> 40	< 13	No
9	Ga08-05	High oil content landrace population	No	None	Medium	> 40	< 13	No
9	Ga09-02	Breeding population for increased oil content	No	None	Medium	> 40	< 13	No
9	Ga10-08	High oil content landrace population	No	None	Early	> 40	< 13	No
10	Ga101B-3	Breeding population for photoperiod insensitivity	No	None	Early	35–40	< 13	Yes
10	Ga101B-5	Breeding population for photoperiod insensitivity	No	None	Early	35–40	< 13	Yes
10	Ga101B-m	Breeding population for photoperiod insensitivity	No	None	Early	35–40	< 13	Yes

*RNA was extracted from more than one plant.

### Planting and sampling

Seeds of the 30 genotypes were planted in 1.5 L plastic pots filled with soil at the Swedish University of Agricultural Sciences (Alnarp, Sweden). Leaf tissue from each genotype was collected one month after planting, snap-frozen in liquid nitrogen, and stored at − 80 °C until RNA extraction was performed.

### RNA extraction and quality control

Total RNA was extracted from approximately 100 mg of leaf tissue from each sample via the RNeasy Plant Mini Kit (74904, QIAGEN). Next, DNase treatment was performed on the extract via an Ambion Turbo DNA-Free Kit (AM1907, Thermo Fisher Scientific, CA, USA). The quantity and quality of the extracted RNA were assessed via an Agilent Bioanalyzer 2100 (Agilent Technologies, CA, USA), a NanoDrop ND-1000 spectrophotometer (Saveen Werner, Sweden), and agarose gel electrophoresis. High-quality RNA samples were subsequently sent to CD Genomics (New York, USA) for RNA sequencing and analysis. Upon arrival, further samples were examined on 1% agarose gels for any evidence of degradation or contamination. The samples were then assessed for purity on a spectrophotometer (IMPLEN, CA, USA), the concentration was measured with a Qubit 2.0 fluorometer (Life Technologies, CA, USA), and sample integrity was assessed using the Agilent Bioanalyzer 2100 system (Agilent Technologies, CA, USA) and the RNA Nano 6000 Assay Kit.

### Library preparation, clustering, and sequencing

The RNA library of each sample was created from 1.5 mg of RNA via the NEBNext UltraTM RNA Library Prep Kit for Illumina (NEB, USA) according to the manufacturer’s instructions. Index codes were added to the adapter sequences to identify different sequences to their respective samples, as described previously by^59^. The library fragments were subsequently cleaned via the AMPure XP system (Beckman Coulter, Beverly, USA), which recognizes fragments of insert sizes 150–200 bp long. Following adapter ligation and PCR amplification, the DNA fragments were purified with Beckman Coulter’s AMPure XP system (Beverly, USA). Library quality was assessed using an Agilent Bioanalyzer 2100 system before clusters and paired-end sequencing were generated on the Illumina HiSeq 2500 platform. The index-coded samples were subsequently clustered via the TruSeq PE Cluster Kit v3-cBot-HS (Illumina) according to the manufacturer’s instructions. Finally, the sequences were clustered, and high-quality paired-end reads were generated using the Illumina HiSeq 2500 platform.

### Data quality control, *de novo* transcript assembly, and splicing

The raw sequencing reads were filtered via a series of methods to obtain high-quality data for further analysis. First, adapters and poly-N sequences of the raw reads were removed via in-house Python scripts, and clean reads were obtained. Then, Phred quality scores were calculated for the clean reads, and reads with Phred quality scores < 30 (error rate > 0.1%) were excluded. The Trinity software package (Trinity v. 2.1.1;^60^ performs *de novo* transcript assembly of high-quality reads since no reference genome is available for noug. Recent studies highlight key considerations for robust *de novo* transcriptome assembly, including quality control metrics and parameter optimization^24,25^. To accomplish this, single-read1 and single-read2 files were created by merging the two read files of the 30 genotypes and then used for transcript assembly and splicing with Trinity^60^utilizing the parameter max_kmer_cov as 2 and all other parameters as defaults. The analysis of the length distribution of transcripts led to the identification of the longest spliced transcripts of different genes, i.e., unigenes, which were used for various downstream analyses. The unigenes have been deposited at DDBJ/EMBL/GenBank as a Transcriptome Shotgun Assembly project under accession number GJSF00000000 (https://www.ncbi.nlm.nih.gov/nuccore/GJSF00000000). The RNA-seq quality-trimmed raw reads were deposited in the Sequence Read Archive (SRA) under accession number PRJNA763316 (https://www.ncbi.nlm.nih.gov/bioproject/PRJNA763316/).

### Gene expression level and differential expression analyses

The gene expression level in each sample was estimated via RNA sequencing via the expectation maximization package (RSEM v.1.2.08;^61^which is based on read counts determined via the mapping of sequenced paired-end reads onto the assembled transcriptome. DEGs were identified between pre-defined phenotypic groups (Table 4) to target specific trait contrasts despite group heterogeneity in other traits. This prioritizes the discovery of trait-associated candidates over strict group-wide expression differences. By calculating fragments per kilobase pair per million reads (FPKM, the abundance of each gene was determined, and transcripts with FPKM values greater than 0.5 were regarded as expressed. The DESeq2^62^ R package was used to perform differential expression analysis of the ten groups of genotypes. While biological replication was limited (pooled RNA for Ga01-12 and Ga01-16; single plants for others), DESeq2’s dispersion estimation (fitType = ‘local’) accounts for unreplicated designs to identify candidate DEGs for downstream validation. A gene was considered significantly differentially expressed if it presented a false discovery rate (FDR)-adjusted *P value* below 0.01 and a log_2_-fold change (log_2_FC) above 2. DEGs and genotype groups were evaluated using a two-way hierarchical cluster analysis with the pheatmap^63^ v.1.0.8 R package. Principal component analysis (PCA) was conducted via the “adegenet”^64^ package in R to determine the overall relationships among the 30 genotypes. The unweighted pair group method with arithmetic mean (UPGMA) cluster analysis was also performed on the same data based on pairwise Euclidean distance via the “vegan”^65^ package in R.

#### Gene function annotation

Gene function annotations of the unigenes were conducted in six major databases to obtain comprehensive gene function information: Universal Protein (UniProt, http://www.ebi.ac.uk/uniprot/;^66^, Nonredundant Protein (NR; https://www.ncbi.nlm.nih.gov/;^67^), Kyoto Encyclopedia of Genes and Genomes (KEGG; http://www.genome.jp/kegg/;^68^, Nucleotide (NT; https://www.ncbi.nlm.nih.gov/), Gene Ontology (GO; http://www.geneontology.org/;^69^ and the plant transcription factor database (PlantTFDB v.3.0, http://planttfdb.gao-lab.org;^70^. The functional annotation of differentially expressed genes (DEGs) was performed via major genomic databases. GO terms were mapped via InterProScan, followed by enrichment analysis with topGO (Fisher’s exact test). Transcription factors were identified through a BLAST search against PlantTFDB v3.0 (E-value < 10^-10, query coverage > 50%, identity > 40%).

### Validation of DEGs by qRT-PCR

Eight DEGs (2 self-compatibility, 2 photoperiod-related, 1 flowering time, 3 oil biosynthesis) were selected for validation using quantitative reverse transcription PCR (qRT-PCR). Total RNA from 18 representative genotypes (3 per group from Groups 2, 7, 8, and 10) was reverse-transcribed using the SuperScript IV First-Strand Synthesis System (Thermo Fisher). Gene-specific primers (Supplementary Table S7) were designed using Primer-BLAST with melting temperatures ranging from 58 °C to 60 °C. Reactions were performed in triplicate on a QuantStudio 3 system using PowerUp SYBR Green Master Mix (Applied Biosystems), with actin as the reference gene. The ΔΔCt values were calculated and compared to RNA-seq log_2_FC values.

## Supplementary Information

Below is the link to the electronic supplementary material.

Supplementary Material 1

Supplementary Material 2

## Data Availability

The data presented in this manuscript are included in supplemental tables, and the raw data were submitted to NCBI under the BioProject ID: PRJNA763316 (https://www.ncbi.nlm.nih.gov/bioproject/PRJNA763316).
