# Supplementary figures and images for: Transcriptome analysis identifies genes regulating self-compatibility, flowering time, and oil biosynthesis in Noug (Guizotia abyssinica)
Source: Sci Rep. 2025 Sep 12;15:32475. doi: 10.1038/s41598-025-18728-x (PMC12432173; doi:10.1038/s41598-025-18728-x)

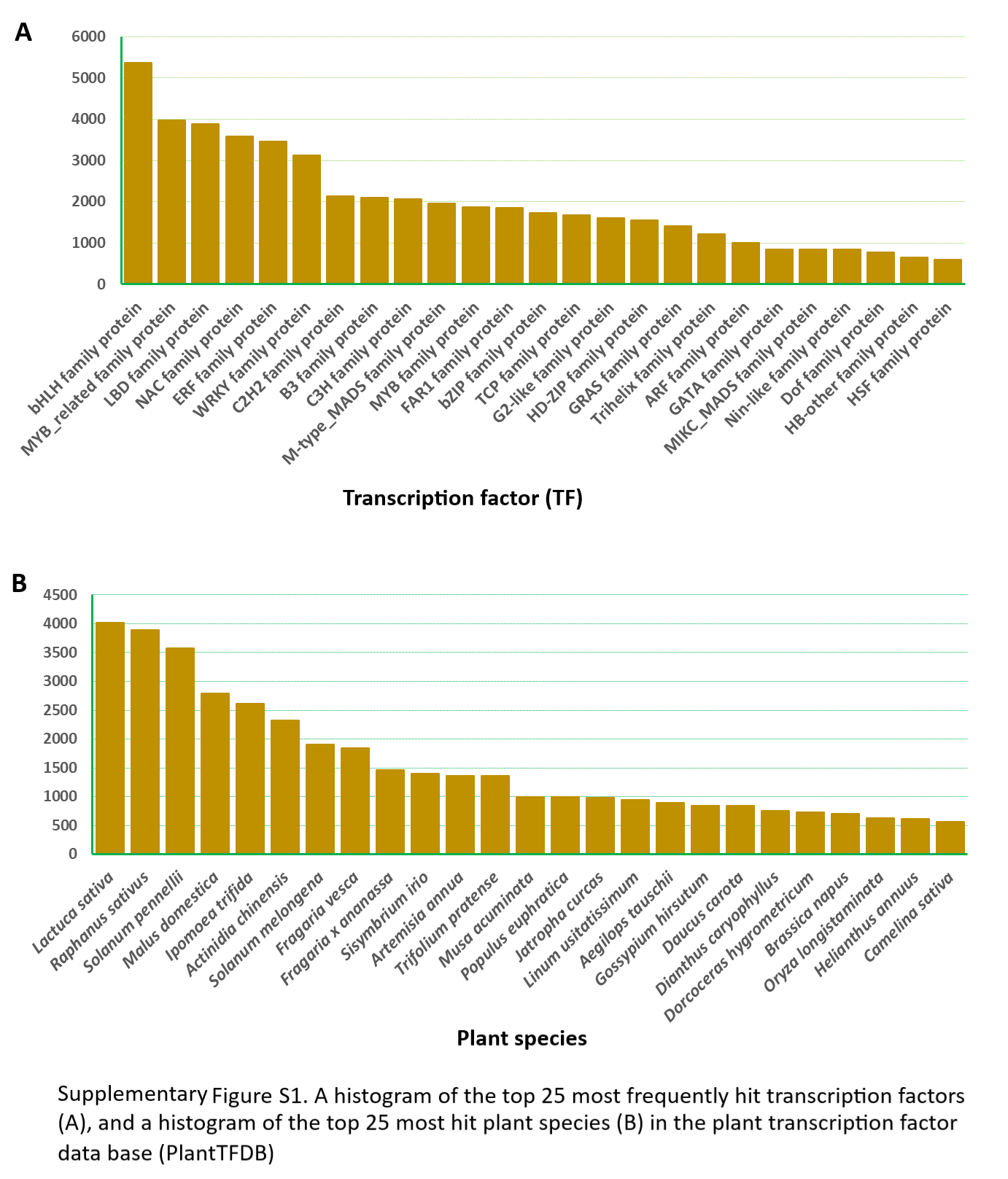

Supplement: Supplementary file 1 — Supplementary Material 1 [file 41598_2025_18728_MOESM1_ESM.tiff]
