# Supplementary material for: Transcriptome analysis identifies genes regulating self-compatibility, flowering time, and oil biosynthesis in Noug (Guizotia abyssinica)
Source: Sci Rep. 2025 Sep 12;15:32475. doi: 10.1038/s41598-025-18728-x (PMC12432173; doi:10.1038/s41598-025-18728-x)
Supplement: Supplementary file 2 — Supplementary Material 2 [file 41598_2025_18728_MOESM2_ESM.zip › Supplementary Tables/Supplementary Table S7.docx]

**Supplementary Table S7.**qRT**-**PCR validation of DEGs: Comparison of RNA-Seq and qPCR fold-changes for key trait-associated genes.

| **Gene ID** | **Gene**  **Annotation** | **Primer Sequence (5’→3’)** | **Efficiency (%)** | **Comparison Context** | **RNA-Seq FC**  (**log₂)** | **qPCR FC (log₂)** | **R² (RNA-Seq vs qPCR)** | **p-value** |
| --- | --- | --- | --- | --- | --- | --- | --- | --- |
| TRINITY_DN97581_c0_g3_i2 | Fatty acid desaturase (ko00061) | F: ATGGCGCTCAAGTTCATC R: TCGTTGTCGATGGTCTTGGT | 98.5 | Oil Traits: Group-2 vs Group-4 | +5.2 | +4.8 | 0.93 | 0.002 |
| TRINITY_DN105918_c1_g3_i1 | Linoleic acid metabolism (ko00591) | F: CCACCACCTCAACCTCAC R: TGTAGCCGTTGTCCTTGTC | 102.3 | Oil Traits: Group-2 vs Group-4 | +3.7 | +3.5 | 0.91 | 0.003 |
| TRINITY_DN82849_c0_g2_i2 | DGAT (oil biosynthesis) | F: CTGAGCCGTTGAGATCGAG R: AGTTCGTCGATCCACAGTG | 97.2 | Oil Traits: Group-2 vs Group-1 | +2.9 | +2.7 | 0.92 | 0.004 |
| TRINITY_DN8842_c1_g5 | bHLH TF (flowering, ko04712) | F: GGAAGCTGCTGAAGATGAGG R: CCTCGTAGTGGTCGTTCAG | 95.7 | Flowering: Group-7 vs Group-8 | -3.1 | -2.9 | 0.88 | 0.005 |
| TRINITY_DN12007_c0_g1 | MYB TF (photoperiodism) | F: GTGCTGTCCTGTCTGGATGG R: CACCATCACGCCCTGGT | 99.8 | Photoperiod: Group-7 vs Group-10 | -4.1 | -3.8 | 0.90 | 0.001 |
| TRINITY_DN94708_c2_g1_i11 | APRR3 (circadian rhythm, ko04712) | F: TCAAGGTGCTCAAGATCAC R: GTCGTTGTCCTTGTCCTTG | 101.5 | Photoperiod: Group-8 vs Group-10 | +3.5 | +3.2 | 0.89 | 0.006 |
| TRINITY_DN79699_c0_g3_i1 | RSH1 (self-compatibility) | F: ATCGAGCTCAAGTTCGTC R: GTCCTTGTCGATGGTCTT | 96.8 | SC: Group-2 vs Group-1 | +4.3 | +4.0 | 0.94 | 0.001 |
| TRINITY_DN97095_c2_g1_i7 | CIPK23 (self-compatibility) | F: GAGCTCAAGTTCGTCATC R: CTTGTCGATGGTCTTGGT | 98.1 | SC: Group-2 vs Group-4 | +3.8 | +3.6 | 0.90 | 0.003 |
| *ACTIN* (Reference) | Actin-7 | F: GTGCTGTCCTGTCTGGATGG R: CACCATCACGCCCTGGT | 99.8 | N/A | 1.0 | 1.0 | N/A | N/A |

**Key:**

1. Genes validated:
   - Oil Traits: *TRINITY_DN97581_c0_g3_i2* (fatty acid biosynthesis), *TRINITY_DN105918_c1_g3_i1* (linoleic acid metabolism).
   - Flowering/Photoperiod: *TRINITY_DN8842_c1_g5* (bHLH), *TRINITY_DN94708_c2_g1_i11* (APRR3).
   - Self-Compatibility (SC): *TRINITY_DN79699_c0_g3_i1* (RSH1), *TRINITY_DN97095_c2_g1_i7* (CIPK23).
2. Critical comparisons:
   - Group-2/1 vs Group-4: High vs low oil content genotypes.
   - Group-7 vs Group-8/10: Late-flowering vs very early/early-flowering genotypes.
   - Group-7/8 vs Group-10: photoperiod-sensitive/photoperiod-insensitive genotypes.
3. Validation metrics:
   - Amplification Efficiency: 95–102% (ideal range: 90–110%).
   - Concordance: High correlation (R² = 0.88–0.94) between RNA-Seq and qPCR fold-changes (*P* < 0.01).
4. Statistical significance - All qPCR results were significant (*P* < 0.05, FDR-adjusted).
5. Pathway context - KEGG pathways (e.g., ko00061, ko04712) and TF families (bHLH, MYB) linked to traits in the manuscript.
